# Human papillomavirus type-specific distribution in cervical intraepithelial neoplasia and cancer in The Gambia prior to HPV immunization programme: a baseline for monitoring the quadrivalent vaccine

**DOI:** 10.1186/s13027-024-00601-7

**Published:** 2024-09-12

**Authors:** Haddy Bah, Foday Ceesay, Ousman Leigh, Haddy Tunkara Bah, Ahmad Tejan Savage, Patrick T. Kimmitt

**Affiliations:** 1 School of Medicine and Allied Health Sciences, University of The Gambia, Independence Drive, Banjul, The Gambia; 2 Department of Laboratory Medicine, Edward Francis Small Teaching Hospital, Independence Drive, Banjul, The Gambia; 3 The American International University, West Africa, Kanifing Institutional Layout, Banjul, The Gambia; 4 School of Life Sciences, University of Westminster, 115 New Cavendish Street, London, W1W 6UW UK

**Keywords:** HPV genotype, Human papilloma virus, Cervical cancer, HPV vaccines, Squamous carcinoma

## Abstract

**Background:**

Cervical cancer is the leading cause of cancer deaths in Gambian women. Current estimates indicate that 286 women are annually diagnosed with cervical cancer with a fatality rate of 70%. In an attempt to address this, in 2019 the quadrivalent HPV vaccine was incorporated into the Gambia’s Expanded Programme on Immunisation. The study aims to retrospectively assess the prevalence and distribution of high-risk HPV genotype in archived, formalin fixed paraffin embedded cervical biopsy tissues diagnosed with cervical cancer in the Gambia from year 2013–2022.

**Method:**

A total of 223 samples with histologically diagnosis of cervical cancer with adequate tissues were sectioned and deparaffinised, followed by HPV DNA extraction and the detection of HR-HPV by real-time multiplex PCR. The human β-globin gene was amplified in 119 samples, which were subsequently tested for HPV DNA.

**Results:**

HPV was prevalent in 87.4% (104 of 119) cervical cancer cases, 12.6% (15/119) samples tested negative. Amongst cervical cancer cases, HPV 16 genotype was the most frequent type accounting for 53.8% (56 /104), followed by other HR-HPV genotypes 17.3% (18/104), and HPV genotype 18 was 15.4% (16/104). Furthermore, multiple HPV infections involving HPV 16 and /or 18 was detected in 14 cases as follows: HPV genotypes 16 and 18 (3.8%, 4 /104), HPV 16 and other HR-HPV (6.7%, 8/104), and HPV 18 and other HR-HPV (1.9%, 2/104). A significant association between age and diagnosis with cervical cancer (*p* = 0.02), and HPV genotype 16 (*p* = 0.04) was observed.

**Conclusion:**

There was no difference in the distribution of HPV 16 and 18 genotypes in cervical cancer cases in The Gambia in comparison with the global distribution. However, the high prevalence of cervical cancer cases with other HR-HPV, and combined infections of HPV 16 with other HR-HPV genotypes seen in this study, clearly shows that the nonavalent HPV vaccine could be more beneficial for The Gambia. This study provides The Gambia with a baseline data to use in policy decisions regarding future evaluation of the quadrivalent HPV vaccine in the country.

## Background

Globally, cervical cancer is the fourth most common cancer among women with over 604,000 new cases and approximately 340,000 deaths estimated to have occurred in 2020 [1]. Additionally, cervical cancer in developing countries accounts for more than 85% of the global estimated cervical cancer deaths [1]. Furthermore, an estimated 110,300 cervical cancer cases have been reported to occur in sub-Saharan African in 2020 [2]. The high cases of cervical cancer observed in developing countries may be attributed to low awareness and limited resources to successfully implement nationwide screening programs. Furthermore, the global mortality rate reported from cervical cancer is almost 10 times greater in the developing countries than in developed countries. This is mainly due to late presentation combined with inadequate access to cancer chemotherapy [3].

In the Gambia, cervical cancer ranks as the most frequent cancer among reproductive aged women 15–44 years with an age standardised incidence rate of 42.9 and age standardised mortality rate of 33.9 per 100,000, respectively [4]. Current estimates indicate that every year 286 women in the Gambia are diagnosed with cervical cancer and approximately 70% die from the disease.

Persistent infection with high-risk human papillomavirus (HR-HPV) genotypes has been identified as the cause for the development of approximately 95% of cervical cancer cases [5]. There are at least 15 HR-HPV genotypes that are known to be associated with cervical cancer, namely: HPV 16,18, 31,33,35,39,45,51,52,53,56,58,59,66 and 68 [6]. In an attempt to reduce the burden of HPV infection, three recombinant HPV prophylactic vaccines have been developed: a bivalent vaccine that targets HR-HPV genotypes16, 18, a quadrivalent vaccine against HR-HPV 16, 18 and low risk (LR) HPV 6 and 11, and Gardasil 9, which targets 7 HR-HPV genotypes 16, 18, 31, 33, 45, 52, 58 and two LR-HPV, 6 and 11. HPV16 and HPV18 genotypes have the strongest association with cervical cancer with international studies reporting their presence in about 70% of cervical cancers [7]. Furthermore, the distribution of other HR-HPV genotypes among cervical cancers across continents and even within Africa has been reported [8–13].

The Gambia is a small country situated in West Africa with a population size of less than 2.5 million; it is divided into namely: Banjul (the capital city). Kanifing Municipal Council (KMC), West Coast Region (WCR), Lower River Region (LRR), North Bank Region (NBR), Central River Region (CRR), and Upper River Region (URR). Both Kanifing Municipal and West Coast Region are closer to the capital city, Banjul. The Gambia has different ethnic groups with Mandinka, Wolof, and Fula being the main ethnic groups The quadrivalent HPV vaccine was introduced in the Gambia in 2019 targeting girls aged 9–14 years residing in the Kanifing Municipality and the Western Region. A vaccine coverage of 83% was achieved for the first dose. However, in 2021, the second dose coverage was about 30%, which was below the expected target.

In the Gambia, studies have shown that the most circulating HR-HPV was HPV 52 and 51 genotypes [14]. Similarly, a study carried out in Senegal, the country that the Gambia only shares border with, also reported HPV 52 and 31 genotypes [16] and in Ghana, HPV 52 and 56 genotypes were reported [17]. However, in Nigeria, HPV 35 and 16 genotypes were reported as the most circulating genotypes [18].

The distribution pattern of HR-HPV for the Eastern and Southern African countries is slightly different. HPV 16 were amongst the most circulating genotypes reported as compared to that of the West African countries. A systematic review and meta-analysis study carried out on sub-Saharan African women showed that HPV 16, 52,18,39, and 31 genotypes are widely distributed in East Africa, whilst HPV 16, 52,18, 56 and 58 genotypes were reported for Southern African countries [19]. Furthermore, the global circulating HR-HPV distribution also varies from that of the African regions as HPV 16 and 18 genotypes are reported, globally [20, 21].

Although, an urban study found that other HR-HPV genotypes are the most circulating HPV genotypes in the Gambia [14], this is the first baseline study characterising HPV genotypes in archived formalin fixed paraffin embedded (FFPE) biopsy cervical cancer tissues. The primary objectives of this study were to retrospectively assess the prevalence of HPV in archived FFPE cervical biopsy tissues with cervical cancer and to determine if the circulating other HR-HPV genotypes are responsible for causing cervical cancers in the Gambia. The study also aims to determine whether there is any difference in the distribution of HR-HPV genotypes in cervical cancerous cases in the Gambia in comparison with the global distribution of HPV 16 and 18 genotypes.

## Method

### Sample selection and preparation

The Edward Francis Small Teaching Hospital (EFSTH) is the only tertiary referral hospital for patients or specimens with any form of suspected cancer in The Gambia. The Pathology department maintains a register where all received and processed tissue samples are recorded. This register was searched to identify all the formalin-fixed paraffin embedded (FFPE) blocks of cervical tissues diagnosed histologically with high grade precursor lesion (CIN III, high grade squamous Intraepithelial lesion (HSIL), or carcinoma in situ) or cervical cancer (adenocarcinoma, adenosquamous carcinoma or any form of squamous cell carcinoma) between January 2013 to December 2022. The retrieved FFPE blocks together with their Haematoxylin & Eosin (H&E) stained slides were examined for cancerous tissues and verified by the department pathologist. FFPE samples that were confirmed to contain adequate cancerous tissues were selected. For H &E slides that were inconclusive, a new section was cut, stained and reviewed.

### Inclusion criteria

All FFPE cervical cancer tissue blocks that are attached to the cassette, properly stored, have adequate tissue material, and have all the relevant information such as age, address, ethnicity, diagnosis stated were included. Once the inclusion criteria were met, patients ‘identifiable data was anonymised using study codes.

### Exclusion criteria

All archived Atypical Squamous Cells of Undetermined Significance (ASCUS), CIN1/2 cases, inadequate tissue material, and those with missing information were excluded. Furthermore, all samples that failed to amplify the human β-globin gene and those with indeterminate results after DNA extraction and analysis were further excluded.

### Tissue sectioning

**Fig. 1:**
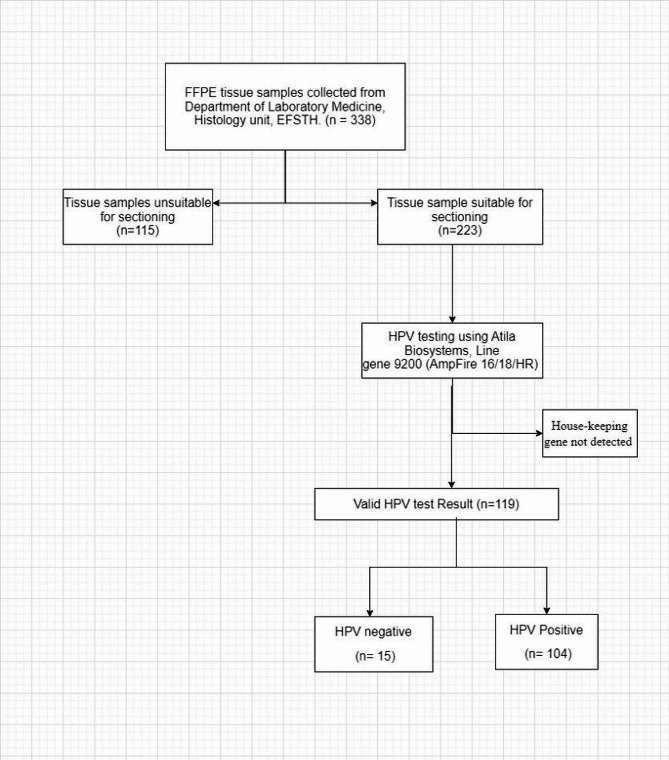
Sample sectioning and human papillomavirus (HPV) testing from archived FFPE tissue blocks, received in Department of Laboratory Medicine, Histology unit, EFSTH

A total of 338 FFPE tissue samples with either HGSIL or cervical cancer were retrieved from the pathology department. Out of the 338 FFPE blocks, 223 were adequate for sectioning (Fig. 1). Prior to sectioning the tissues, the microtome stage and forceps were cleaned with xylene followed by ethanol, to avoid cross-contamination from other specimens. For each FFPE block, a fresh blade and a new container of water for floating sections was used. Sixty (60 μm) micron of tissue samples were sectioned from each FFPE block and placed into their corresponding labelled 1.5 mL Eppendorf tube for DNA extraction.

### HPV DNA extraction

The human papillomavirus nucleic acid was extracted from the FFPE samples using the AmpFire HPV Screening kit (Atila Biosystems). Briefly, the extraction process consists of lysing of the 60 μm tissue samples with 150 mL solution A (Catalog number PRMVR − 10), the tube was vortexed for 30 s to dissolve the paraffin. Fifty (50 µL) microlitre of 1x lysis buffer was added (1x lysis buffer was prepared according to the manufacturer’s guideline). The tubes were vortexed for 10 s to mix prior to spinning to obtain optimal sample lysis. The tubes were incubated in a 95^O^C dry heat block for 1 h 30 min with a brief spun after the first 45 min of incubation. After the incubation, the tubes were allowed to cool at room temperature, vortexed and briefly spun. The supernatant containing the purified nucleic acid was transferred into sterile labelled microtubes for HPV detection and genotyping. The extraction process was quality controlled by including molecular grade water in the extraction process to serve as template extraction blank control in each batch of samples extracted.

### HPV detection and genotyping

HPV detection was carried out using AmpFire HPV 16/18/HR assay (Atila Biosystems, Line gene 9200). It allows simultaneous identification of HPV 16 and 18 and grouped the rest of the HR-HPV (31, 33, 35, 39, 45, 51, 52, 53, 56, 58, 59, 66, 68) as other HR-HPV. The AmpFire test system uses HR- HPV specific primers and fluorescent probes that amplifies regions of the viral genomic DNA including regions of the late (L1) and the early (E6/E7) gene proteins under isothermal conditions. The multiple proteins detected by the system helps to improve the sensitivity of the test. The kit comprises the Reaction Mix (with buffer, enzymes, and dNTPs), Primer Mix (with primers and probes), Positive and Negative Controls. Each assay includes a negative and positive control to establish the quality of the assay performance and to rule out contamination.

The Mastermix reaction was prepared according to the manufacturer’s guideline, and this was well mixed after preparation. Twenty (20 µL) microlitre of the mastermix reaction was dispensed into each labelled PCR tubes. 5 µL of the extracted DNA collected from the bottom layer was transferred to the corresponding PCR tubes. Five (5 µL) microlitre of the test kit Positive and Negative Controls were added to their corresponding labelled PCR reaction tubes, respectively. In addition to the test kit Positive and Negative Controls, a molecular grade water was tested as an in-house Negative Control. All the reaction tubes were capped and gently vortexed to mix all the reagents, followed by a brief spun to bring down all liquid to the bottom of the wells. The PCR tubes were subsequently placed into the sample holder in the Atila Biosystem real-time PCR machine, and the reaction was run at 60^o^C for 75 min. The results were interpreted based on real-time fluorescent detection of the specific labelled probes CY5, ROX, FAM, and HEX for HPV16, HPV18, other HR-HPV, and the human β-globin gene (internal control, IC), respectively. Samples that failed to amplify the human β-globin gene were classified as inadequate or invalid test result. All inadequate and HPV negative samples were rescreened twice using a new sectioned tissue.

### Data analysis

Data was entered into the Epi-Info version 7 (https://www.cdc.gov/epiinfo) software and analysed using IBM SPSS version 21.0. Analysis was primarily descriptive with proportions of HPV-16, 18, and other HR-HPV types detected in the cancer tissues. Categorical variables were expressed in frequencies and percentages. Fisher’s Exact test was used to determine association between age and cervical cancer diagnosis, and the different HPV genotypes variables at a 95% confidence interval (CI) and *p-value of ≤ 0.05* were considered statistically significant.

## Results

The mean age of the 338 women that samples were collected from was 50.4 (SD ± 12.6, range 13–85) years. Out of the 338, 326 (96.4%) of them were diagnosed with cervical cancer; 300 (92%) of these cancers were squamous cell carcinoma, 14 (4.3%) adenocarcinoma, 11 (3.4%) carcinoma in-situ and 1 (0.3%) with CIN III with high degree of invasive suspicious of invasive carcinoma.

Of the 119 samples that were adequate for HPV DNA screening, 104 were HPV positive and 15 were HPV negative, thus giving HPV positivity rate of 87.4% (104/119). All infections involving HPV 16 and HPV 18 genotypes, as single infections, accounted for 69.2% of the cervical cancer cases. As single infections, HPV 16 accounted for 53.8% (56/104) of the HPV positive cervical tissues, and HPV 18 accounts for 15.4% (16/104), (Table 1). All the remaining 13 HPV genotypes grouped together as other HR-HPV accounts for 17.3% (18/104). Furthermore, Multiple infections involving HPV 16 and/or 18, were as follows: HPV16 and 18 genotypes (3.8%, 4/104), HPV genotype 16 and other HR-HPV (6.7%, 8/104), HPV 18 and other HR-HPV (1.9%, 2/104).

**Table 1:** Distribution of HR-HPV genotypes in histologically diagnosed cervical cancer cases and patients’ age

	Squamous cell carcinoma		Adenocarcinoma		Carcinoma in-situ		Total	
	HPV positive cases (n)	HPV Prevalence (%)	HPV positive cases (n)	HPV Prevalence (%)	HPV positive cases (n)	HPV Prevalence (%)	HPV positive cases (n)	HPV Prevalence (%)
All	97/104	93.3	5/104	4.8	2/104	1.9	104/119	87.4
**Age (years)**								
21–31	3/97	3.1	0	0	0	0	3/104	2.9
32–42	27/97	27.8	1/5	20.0	2/2	100	30/104	28.8
43–53	28/97	28.8	1/5	20.0	0	0	29/104	27.9
54–64	23/97	23.7	1/5	20.0	0	0	24/104	23.1
65–75	14/97	14.4	1/5	20.0	0	0	15/104	14.4
76–86	2/97	2.1	1/5	20.0	0	0	3/104	2.9
**HPV type**								
16	53	54.6	1	20.0	2	1.9	56	53.8
18	14	14.4	2	40.0	0	0	16	15.4
16 /18	4	4.1	0	0	0	0	4	3.8
16/others-HR-HPV	7	7.1	1	20.0	0	0	8	6.7
18/ others-HR-HPV	1	1.0	1	20.0	0	0	2	1.9
Others-HR-HPV	18	18.6	0	0	0	0	18	17.3

A majority 93% (97/104) of the HPV positive cervical samples were diagnosed with squamous cell carcinoma and had HPV 16 as the more prevalent genotype (54.6%, 53/97), followed by other HR-HPV (18.6%, 18/97), and HPV 18 accounting for 14.4%, (14/97) of the squamous cell carcinoma cases, respectively (Table 1).

HPV distribution amongst the different age groups showed HPV 16 related cancer was more prevalent among 43 − 53-year-old (18.3%), followed by 32–42-year aged group (14.4%) (Table 3). The results also showed that 39% of the cervical cancer biopsies that were HPV positive were from the Mandinka ethnicity, followed by the Jola (20%). The distribution of cervical cancer cases in the different regions of the country showed that the majority of cases received for the study, 46.2% (156/338) were from the West Coast Region (WCR) and 30.7% (104/338) from KMC. Fewer cervical cancer cases were recorded for regions that are further away from the EFSTH, Pathology Department; 7.1% (24/338) North Bank, 5.1% (17/338) Lower River, 3.6% (12/338) Central River and 4.1% (14/338) for Upper River (Table 2). Of samples with adequate DNA from these regions, the proportion of HR-HPV in descending order were as follows: 50.9% (53/104) for WCR, 28.8% (30/104) KMC, 6.7% (7/104) LRR, 5.8% (6/104) NBR, 2.9% (3/104) URR, 2.9% (3/104) Banjul, and 1.9% (2/104) CRR, respectively (Table 2).

**Table 2:** Distribution of HR-HPV amongst cervical cancer cases in the different regions of The Gambia

Region	**Total number of cervical cancer cases**	**Total number with adequate DNA**	**Total HR-HPV Positive**	**Total HR-HPV Negative**
Banjul	11 (3.2)	4 (3.4)	3 (2.9)	1 (6.7)
KMC	104 (30.7)	32 (26.9)	30 (28.8)	2 (13.4)
WCR	156 (46.2)	53 (44.5)	53 (50.9)	0 (0.0)
NBR	24 (7.1)	17 (14.3)	6 (5.8)	11 (73.3)
LRR	17 (5.1)	7 (5.9)	7 (6.7)	0 (0.0)
CRR	12 (3.6)	2 (1.7)	2 (1.9)	0 (0.0)
URR	14 (4.1)	4 (3.4)	3 (2.9)	1 (6.7)
Total	**338**	**119**	**104**	**15**

KMC - Kanifing Municipal Council; WCR – West Coast Region; NBR – North Bank Region; LRR – Lower River Region; CRR – Central River Region; URR – Upper River Region

A cross-tabulation analysis showed there was a significant association between participants’ age with their diagnosis with cervical cancer (*p* = 0.02) and HPV genotype 16 (*p* = 0.04) (Table 3). However, no significant association between ethnicity (*p* = 0.62), residential area (*p* = 1.00) of the participants and their diagnosis with cervical cancer, HPV result and HPV genotype were observed. The participants within the ages 43–53 years had the highest frequency (*n* = 100, 29.6) of cervical cancer diagnosis and with HPV Genotype 16 (*n* = 19, 18.3%) as the most prevalent type. Fewer HPV positive cervical cancers were observed in the older age group (76–86 years) (Table 3).

**Table 3:** Association between Age, HPV genotype and cervical Cancer

Variable	Age in years *n* (%)						Fisher’s exact test	*P*-value
	**21–31**	**32–42**	**43–53**	**54–64**	**65–75**	**76–86**		
Diagnosis (*n* = 338)							11.4	0.023*
Cervical Cancer	11 (3.3)	82 (24.3)	100 (29.6)	79 (23.4)	45 (13.3)	9 (2.7)		
Not Cervical Cancer	1 (0.3)	4 (1.2)	3 (0.9)	3 (0.9)	-	-		
HPV Result (*n* = 119)							2.4	0.790
Positive	3 (2.5)	30 (25.2)	29 (24.4)	24 (20.2)	15 (12.6)	3 (2.5)		
Negative	-	3 (2.5)	6 (5.0)	3 (2.5)	2 (10.5)	1 (0.8)		
HPV Genotype (*n* = 104)								
HPV 16	1 (0.9)	15 (14.4)	19 (18.3)	10 (9.6)	11 (10.6)	-	16.04	0.041*
HPV18	1 (0.9)	7 (6.7)	2 (1.9)	4 (3.8)	2 (1.9)	-	4.8	0.564
HPV 16 /18	-	1 (0.9)	2 (1.9)	1 (0.9)	-	-	1.3	0.971
HPV 16 /other HR-HPV	-	-	4 (4.8)	2 (1.9)	1 (0.9)	1 (0.9)	6.41	0.379
HPV 18 / other HR-HPV	-	-	-	1 (0.9)	-	1 (0.9)	18.95	0.004*
Other HR-HPV	1 (0.9)	7 (6.7)	2 (1.9)	6 (5.8)	1 (0.9)	1 (0.9)	6.06	0.416

## Discussion

Persistent infection with high -risk HPV genotypes has been identified as the most important aetiologic agent in the pathogenesis of cervical cancer [22]. Although, geographical variations in the prevalence of HPV DNA in cervical cancer biopsies, has been reported worldwide, the overall prevalence has been reported to be approximately 95% [23, 24]. In this cross-sectional study of women diagnosed with cervical cancer from 2013 to 2022, HPV prevalence was found to be 87.4% in the analysed cervical cancer tissues with HPV 16 genotype being the most frequent type detected (53.8%), followed by other HR-HPV genotype (17.3%), and HPV 18 genotype accounting for (15.4%) (Table 1). In addition, the combined prevalence of HPV 16 and 18 genotypes accounts for approximately 70% of the cervical cancer cases diagnosed in this study. However, a low HPV 16 carriage rate was observed in cervical swabs from reproductive aged women from Bah Camara et al. (2018) study [14]. Their study identified other HR-HPV genotypes as the most common circulating type in urban Gambia. The high HPV prevalence rate of 87.4% found in this study is similar to other studies conducted in the neighbouring country, Senegal (90.0%), and in Nigeria (90.7%) [25, 26]. In addition, HPV studies carried out on FFPE samples in Malawi and South Africa also reported a high prevalence rate of 97.0% and 92.0%, respectively [27, 28]. However, countries including Poland and Iran reported a lower HPV prevalence rate in cervical cancer [29, 30]. These differences in HPV prevalence in cervical cancer could be attributed to several factors which may include geographical variations, quality and quantity of samples, sensitivity and specificity of the methods used for DNA extraction and HPV detection [31, 32].

The high prevalence (53.8%) of HPV16 reported in this FFPE study was consistent with other studies, as this genotype is the most prevalence type reported, globally [33–36]. Epidemiological, clinical, and molecular studies have shown that HPV 16 and HPV18 genotypes are combinedly responsible for almost 70% of cervical cancers [36, 37]. The combined prevalence rate (69.2%) of HPV 16 (53.8%) and HPV 18 (15.4%) genotypes reported in this retrospective cross-sectional study corresponds to the estimate of the global distribution of these genotypes responsible for causing cervical cancers, worldwide. They are the most prevalent and most potent carcinogenic viruses, and their probability of disease progression and persistence is significantly higher than other high risk HPV genotypes [38]. Furthermore, in the 97 tissues diagnosed with squamous carcinoma, HPV 16 genotype accounts for 54.6% (53/97) (Table 1). This result agrees with that of Wall et al., carried out in the Gambia [15] and Missaoui et al., carried out in Tunisia, North Africa [39]. Both studies found that HPV 16 is the most frequent genotype among invasive squamous cell carcinomas. However, some studies have reported that other HR-HPV genotypes other than HPV 16 are most common HPV type detected in cervical cancer [40, 41]. A study carried out in Parakou, Benin Republic, reported that HPV 16 genotype was not detected in any of the cervical cancer cases, and HPV 39 was the most common genotype detected in their study [41]. This could be due to geographical variation and /or genotype specific replacement as 15% of cervical cancers are reported to be caused by other HR-HPV [42].

An observation in this study was the high proportion (17.3%) of cervical cancers with other HR-HPV genotypes and (6.7%) combined infections of HPV 16 with other HR-HPV genotypes found in the cervical cancer tissue samples. This finding agrees with the result from Bah Camara et al., [14] study that found other HR-HPV genotypes in cervical samples of HIV positive women diagnosed with cervical cancer in The Gambia [43]. Other studies have also shown that apart from HPV 16 and 18 genotypes, other HR-HPV genotypes that the quadrivalent HPV vaccine does not offer protection against causes cervical cancers [42]. Although, the Gambia is currently administering the quadrivalent vaccine which targets HPV genotypes 6, 11, 16 and 18, there is a need to switch to the nonavalent HPV vaccine which targets an additional 5 other HR-HPV genotypes (HPV 31, 33, 45, 52, and 58) which are associated of causing 15% of cervical cancers, globally [42].

HPV DNA negative results was found in 12.6% (15/119) histologically diagnosed cervical cancer cases. This could be due to the rare cases of non-HPV related cervical cancer or integration of the viral genome into the host chromosome, which could lead to changes in the genes the PCR primers target.

Of the 338 cervical cancer cases histologically diagnosed in The Gambia, cervical cancer was most common in 43–53-year aged women (29.6%, 100/326). However, HPV related cervical cancer was more prevalent in the 32–42 year (28.8%, 30/104), followed by 43–53 year (27.9%, 29/104) aged women. This finding further shows that women that are mostly affected with cervical cancer in the Gambia are in their mid-adult lives (32–53 years) and possibly having young families. A significant association was found between cervical cancer diagnosis and age (*p* = 0.02) and HPV genotype16 (*p* = 0.04) (Table 3). Although, no significant association was found between HPV genotype, cervical cancer diagnoses and ethnicity (*p* = 0.62), HPV related cervical cancer was mostly detected (39.4%) in the Mandinka ethnicity. However, in Bah - Camara et al., [14] study, circulating HPV was found to be more prevalent (31.3%) in the Fula ethnicity, whereby HPV related cervical cancer was found to be 16.3% in the Fula ethnicity in this study. The disproportionate distribution of HPV related cervical cancer in the different ethnicity could be multifaceted, which could include late access to early screening, treatment and management of precancerous lesions. Another interesting finding from this study was that less histologically diagnosed cervical cancer cases was observed for the Northern, Central, and Upper Regions of the country compared to the Western (50.9%) and (28.8%) Kanifing Municipal Regions. These differences could be attributed to several factors among which include access, and proximity of the only Histopathology department in the country, which is situated at the teaching hospital in Banjul, the capital city. The low (3.2%, 11/338) histologically diagnosed cervical cancer cases observed in the capital city could also be attributable to residents having access to early cervical cancer screening and timely pre cancer management, which is also offered in the teaching hospital in Banjul. This highlight the need to decentralise cervical cancer screening in the country for easy access and management of precancerous lesions, and prompt treatment options.

## Conclusion

This study showed that the overall frequency of HPV genotypes detected in women with cervical cancer in The Gambia was high. HPV 16, other HR-HPV, and HPV 18 genotypes were responsible for causing cervical cancer. The combination of HPV 16 and other HR-HPV genotypes were also observed. In view of these results, the use of the Gardasil-9^®^ (nonavalent) vaccine could provide a more effective cervical cancer prevention in The Gambia. Furthermore, monitoring the changes in HPV genotype distribution will allow ongoing assessment of the impact of the quadrivalent vaccine and potential, but unlikely, type replacement. This baseline data will help policy makers and implementers in policy decisions regarding future evaluation of the quadrivalent HPV vaccine in the Gambia. The data will also contribute to the literature on other HR-HPV genotypes causing cervical cancers in Africa and other continents.

### Study limitations

One of the study limitations is failure to amplify the human β-globin gene in a high number of FFPE samples. This could be attributed to the long storage of the samples. Some studies have elucidated difficulties in deparaffinising and extracting DNA from long storage FFPE samples. Another limitation encountered in this study was the poorly embedded tissues, not attached on the cassette or had little embedded tissues, which made tissue sectioning impossible. These samples were excluded from the study; therefore, the proposed sample size was not achieved. Furthermore, future work is needed to determine the genotype specific of the other HR-HPV detected in the cervical cancer FFPE tissues.

## Data Availability

Not applicable.

## References

[1] Sung H, Ferlay J, Siegel RL, Laversanne M, Soerjomataram I, Jemal A, et al. Global cancer statistics 2020: GLOBOCAN estimates of incidence and mortality worldwide for 36 cancers in 185 countries. CA Cancer J Clin. 2021;71:209–49. 33538338 10.3322/caac.21660

[2] Bray F, Parkin DM, on behalf of the African Cancer Registry Network. Cancer in sub-saharan Africa in 2020: a review of current estimates of the national burden, data gaps, and future needs. Lancet Oncol. 2022. 10.1016/S1470-2045(22)00270-4. 35550275 10.1016/S1470-2045(22)00270-4

[3] Stelzle D, Tanaka LF, Lee KK, et al. Estimates of the global burden of cervical cancer associated with HIV. Lancet Glob Health. 2020;20:30459–9. 10.1016/S2214-109X(20)30459-9 PMC7815633 33212031

[4] IOC/IARC: Gambia Human papillomavirus and related cancers, Fact sheet. 2023; https://hpvcentre.net/statistics/reports/GMB_FS.pdf

[5] World Health Organization: Cervical Cancer, fact sheet. 2024; https://www.who.int/news-room/fact-sheets/detail/cervical-cancer

[6] IARC (International Agency for Research on Cancer). A review of human carcinogens. Part B: Biological agents/IARC Working Group on the Evaluation of Carcinogenic Risks to humans. Human papillomaviruses, IARC monographs on the evaluation of carcinogenic risks to humans. 2011;100B:255–313.

[7] de Martel C, Plummer M, Vignat J, et al. Worldwide burden of cancer attributable to HPV by site, country, and HPV type. Int J Cancer. 2017;141:664–70. 28369882 10.1002/ijc.30716 PMC5520228

[8] Smith JS, Lindsay L, Hoots B, et al. Human papillomavirus type distribution in invasive cervical cancer and high-grade cervical lesions: a meta‐analysis update. Int J Cancer. 2007;121:621–32. 17405118 10.1002/ijc.22527

[9] Clifford GM, Tully S, Franceschi S. Carcinogenicity of human papillomavirus (HPV) types in HIV-positive women: a meta-analysis from HPV infection to cervical cancer. Clin Infect Dis. 2017;64:1228–35. 28199532 10.1093/cid/cix135 PMC5399941

[10] Akakpo PK, Imbeah EG, Ulzen-Appiah K, Darkwa-Abrahams A. The distribution of hr HPV genotypes among cervical cancer cases diagnosed across Ghana: a cross-sectional study. BMC Infect Dis. 2024;24(1):356. 10.1186/s12879-024-09166-7. 38539128 10.1186/s12879-024-09166-7 PMC10967043

[11] Abba K, Mwajim B, Haruna AN, Harun BR. Prevalence of human papillomavirus genotypes in cervical cancer in Maiduguri, Nigeria. Pan Afr Med J. 2019;33:284. 10.11604/pamj.2019.33.284.18338. 31692869 10.11604/pamj.2019.33.284.18338 PMC6815519

[12] Niane K, Diagne Diop CT, Dia G, et al. Human papilloma virus genotypes associated with cervical cancer in Senegal. Biomed Res Rev. 2021;5:1–6.

[13] Kuguyo O, Dube Mandishora RS, Thomford NE, Makunike-Mutasa R. High-risk HPV genotypes in Zimbabwean women with cervical cancer: comparative analyses between HIV-negative and HIV-positive women. PLoS ONE. 2021;16. 10.1371/journal.pone.0257324 PMC8478215 34582476

[14] Bah Camara H, Anyanwu M, Wright, Kimmitt PT. Human papillomavirus genotype distribution and risk factor analysis in reproductive age women in urban Gambia. J Med Microbiol. 2018;67:1645–54. 30299238 10.1099/jmm.0.000848

[15] Wall SR, Scherf CF, Morison L, et al. Cervical human papillomavirus infection and squamous intraepithelial lesions in rural Gambia, West Africa: viral sequence analysis and epidemiology. Br J Cancer. 2005;93:1068–76. 16106268 10.1038/sj.bjc.6602736 PMC2361674

[16] Mbaye EHS, Gheit T, Dem A, Mckay-Chopin S. Human papillomavirus infection in women in four regions of Senegal. J Med Virol. 2014;86:248–56. 24026804 10.1002/jmv.23719

[17] Donkoh ET, Asmah RH, Agyemang-Yeboah F, Dabo EO. Prevalence and distribution of vaccine-preventable Genital Human Papillomavirus (HPV) genotypes in Ghanaian women presenting for screening. Cancer Control. 2022;29. 10.1177/10732748221094721. 10.1177/10732748221094721 PMC9096183 35536890

[18] Okolo C, Franceschi S, Adewole I, Thomas JO. Human papillomavirus infection in women with and without cervical cancer in Ibadan, Nigeria. Infect Agent Cancer. 2010;5:1–4. 10.1186/1750-9378-5-24. 21129194 10.1186/1750-9378-5-24 PMC3017010

[19] Ayichew S, Nega A, Tadesse G, Berhanu S. Prevalence and genotype distribution of high-risk human papillomavirus infection among sub-saharan African women: a systematic review and Meta-analysis. Front Public Health. 2022;10:1–12. 10.3389/fpubh.2022.890880. 10.3389/fpubh.2022.890880 PMC9304908 35875040

[20] Guan P, Howell-Jones R, Bruni L, de Sanjose S. Human papillomavirus types in 115,789 HPV-positive women: a meta-analysis from cervical infection to cancer. Int J Cancer. 2012;131:2349–59. 10.1002/ijc.27485. 22323075 10.1002/ijc.27485

[21] Li N, Franceschi S, Howell-Jones R, Snijders PJ. Human papillomavirus type distribution in 30,848 invasive cervical cancers worldwide: variation by geographical region, histological type and year of publication. Int J Cancer. 2011;128:927–35. 10.1002/ijc.25396. 20473886 10.1002/ijc.25396

[22] Witkiewicz AK, Wright TC, Ferenczy A, Ronnett BM, Kurman RJ. Carcinoma and other tumours of the Cervix, Blaustein’s Pathology of the Female Genital Tract. Springer. 2011;254–95.

[23] Anna-Barbara M. Natural history of HPV infection in adolescents and relationship to Cervical Cancer, Molecular Pathology of Gynaecologic Cancer. Totowa, New Jersey: Humana. Springer.; 2007. pp. 103–12.

[24] Castellsague X. Natural history and epidemiology of HPV infection and cervical cancer. Gynecol Oncol. 2008;110(3 Suppl 2):S4–7. 18760711 10.1016/j.ygyno.2008.07.045

[25] Okolo C, Franceschi S, Adewole I, Thomas JO, et al. Human papillomavirus infection in women with and without cervical cancer in Ibadan, Nigeria. Infect Agent Cancer. 2010;5:24. 21129194 10.1186/1750-9378-5-24 PMC3017010

[26] Howitt BE, Herfs M, Tomoka T, Kamiza S, et al. Comprehensive Human Papillomavirus genotyping in cervical squamous cell carcinomas and its relevance to Cervical Cancer Prevention in Malawian Women. J Glob Oncol. 2017;3:227–34. 28717764 10.1200/JGO.2015.001909 PMC5493214

[27] Denny L, Adewole I, Anorlu R, Dreyer G, Moodley M, Smith T, et al. Human papillomavirus prevalence and type distribution in invasive cervical cancer in sub-saharan Africa. Int J Cancer. 2014;134:1389–98. 23929250 10.1002/ijc.28425

[28] Dybikowska A, Licznerski P, Podhajska A. HPV detection in cervical cancer patients in northern Poland. Oncol Rep. 2002;9(4):871–4. 12066224

[29] Mahmoodi P, Motamedi H, Abad Shapouri MRS, Shehni MB, Kargar M. Molecular detection and typing of human papillomaviruses in paraffin-embedded cervical Cancer and precancer tissue specimens. Iran J Cancer Prev. 2016;9:e3752. 27366309 10.17795/ijcp-3752 PMC4922202

[30] Biedermann K, Dandachi N, Trattner M, Vogl G, Doppelmayr H, Moré E, et al. Comparison of real-time PCR Signal-amplified in situ hybridization and conventional PCR for detection and quantification of human papillomavirus in Archival Cervical Cancer tissue. J Clin Microbiol. 2004;42:3758–65. 15297527 10.1128/JCM.42.8.3758-3765.2004 PMC497646

[31] Garland SM, Tabrizi S. Methods for HPV Detection: polymerase chain reaction assays, emerging issues on HPV infections, from Science to Practice. Basel Karger. 2006; 63–72.

[32] de Sanjose S, Quint WG, Alemany L, Geraets DT, Klaustermeier JE, et al. Retrospective International Survey and HPV Time trends Study Group. Human papillomavirus genotype attribution in invasive cervical cancer: a retrospective cross-sectional worldwide study. Lancet Oncol. 2010;11:104. 20952254 10.1016/S1470-2045(10)70230-8

[33] Li N, Franceschi S, Howell-Jones R, Snijders PJ, Clifford GM. Human papillomavirus type distribution in 30,848 invasive cervical cancers worldwide: variation by geographical region, histological type, and year of publication. Int J Cancer. 2011;128:927–35. 20473886 10.1002/ijc.25396

[34] Lagheden C, Eklund C, Lamin H, Kleppe SN, Lei J, et al. Nationwide comprehensive human papillomavirus (HPV) genotyping of invasive cervical cancer. Br J Cancer. 2008;118:1377–81. 10.1038/s41416-018-0053-6 PMC5959883 29559733

[35] Liao L, Cheng H, Zeng F, Zhou W, Ding Y. Prevalence and distribution of human papillomavirus genotypes among women with high-grade squamous intraepithelial lesion and invasive cervical cancer in Ganzhou, China. J Clin Lab Anal. 2019;33:e22708. 30390349 10.1002/jcla.22708 PMC6818556

[36] International Agency for Research on Cancer (IARC). Handbooks of cancer prevention Vol 10: cervix cancer screening. 2005.

[37] Muñoz N, Castellsagué X, Berrington de González A, Gissmann L. Chap. 1: HPV in the etiology of human cancer. Vaccine. 2006;24:1–10. 16949995 10.1016/j.vaccine.2006.05.115

[38] Salazar KL, Zhou HS, Xu J, Peterson LE, Schwartz MR, Mody DR, et al. Multiple human papillomavirus infections and their impact on the development of high-risk cervical lesions. Acta Cytol. 2015;59:391–8. 26674365 10.1159/000442512

[39] Missaoui N, Hmissa S, Trabelsi A, Tahar Yacoubi M, Nouira A, et al. Prevalence of HPV infection in precancerous and cancerous lesions of the uterine cervix in Tunisia. Ann Biol Clin (Paris). 2010;68:297–303. 20478773 10.1684/abc.2010.0431

[40] Awua AK, Sackey ST, Osei YD, Asmah RH, Wiredu EK. Prevalence of human papillomavirus genotypes among women with cervical cancer in Ghana. Infect Agents Cancer. 2016;11:4. 10.1186/s13027-016-0050-4 PMC4727324 26816527

[41] Zohoncon TM, Quedraogo TC, Brun LVS, Obiri-Yeboah D, Djigma WF, Kabibou S, et al. Molecular Epidemiology of High-Risk Human Papillomavirus in High-Grade Cervical Intraepithelial Neoplasia and in Cervical Cancer in Parakou, Republic of Benin. Pak J Biol Sci. 2016;19:49–56. 29023039 10.3923/pjbs.2016.49.56

[42] IARC Handbooks of Cancer Prevention. 2022; vol 18: https://publications.iarc.fr

[43] Bah Camara H, Anyanwu M, Mattiuzzo G, Gillard L, Wright E, Kimmitt PT. Human papillomavirus sero-prevalence and sexual attitudes amongst a cohort of HIV positive women in the Gambia. ASM Microbe: WestminsterResearch; 2018b.

